# Recruiting people living with mild cognitive impairment into a fall prevention trial

**DOI:** 10.1186/s13063-026-09533-6

**Published:** 2026-02-10

**Authors:** Elissa Burton, Nicola T. Lautenschlager, Kathryn A. Ellis, Anne-Marie Hill, Meggen Lowry, Rachael Moorin, Joanne McVeigh, Angela Jacques, Kirk I. Erickson, Joel Tate, Sarah Bernard, Carolyn F. Orr, Luke Bongiascia, Melanie L. Clark, Keith D. Hill

**Affiliations:** 1 Curtin School of Allied Health, Faculty of Health Sciences, Curtin University, Perth, WA 6845 Australia; 2 Brightwater Care Group, Inglewood, WA Australia; 3 enAble Institute, Faculty of Health Sciences, Curtin University, Perth, Australia; 4 Department of Psychiatry, The University of Melbourne, Melbourne, Australia; 5 Royal Melbourne Hospital Mental Health Services, Royal Melbourne Hospital, Parkville, VIC Australia; 6 Melbourne School of Psychological Sciences, University of Melbourne, Melbourne, Australia; 7 School of Allied Health, Western Australian Centre for Health and Ageing, The University of Western Australia, Perth, WA Australia; 8 Next Step Health, Brisbane, QLD Australia; 9 Curtin School of Population Health, Faculty of Health Sciences, Curtin University, Perth, Australia; 10 School of Population and Global Health, The University of Western Australia, Perth, Australia; 11 Movement Physiology Laboratory, School of Physiology, University of Witwatersrand, Johannesburg, South Africa; 12 Institute for Health Research, University of Notre Dame Australia, Fremantle, WA Australia; 13 AdventHealth Research Institute, Neuroscience, Orlando, FL USA; 14 Department of Rehabilitation and Aged Care, Armadale Health Service, Perth, Australia; 15 Geriatric Acute and Rehabilitation Medicine, Sir Charles Gairdner Osborne Park Healthcare Group, Perth, Australia; 16 Cognitive Clinic, Royal Perth Hospital, Perth, Australia; 17 Physiotherapy Department, Adult Community and Allied Health Directorate, Rockingham Peel Group, Rockingham, Australia; 18 Neurosciences Unit, North Metropolitan Health Service Mental Health, Public Health and Dental Services, Perth, Australia; 19 Rehabilitation, Ageing and Independent Living (RAIL) Research Centre, School of Primary and Allied Health Care, Monash University, Melbourne, Australia

**Keywords:** Mild cognitive impairment, Recruitment, Randomised controlled trial, Falls, Fall prevention

## Abstract

**Background:**

People with mild cognitive impairment (MCI) fall as often as people living with early dementia. Yet, few trials have investigated the effectiveness of fall prevention interventions for people with MCI. Recruitment of specific populations into long-term trials can be challenging, and to assist future studies, it is necessary to better understand more about trial recruitment. This study outlines the recruitment of people with MCI into the Balance on the Brain trial. This was a single-blind randomised controlled trial based in the Perth Metropolitan Area, which included people living with MCI in the community, aged 50 or over, and who were self-reported to not be meeting the Australian physical activity guidelines. The multi-modal exercise intervention was for 6 months, and all participants were followed up monthly for 12 months. A sample size of 396 was calculated to meet the requirements for the falls primary outcome (212 participants for the balance primary outcome).

**Methods:**

Eleven recruitment strategies were used across the 36-month recruitment (January 2021 through to December 2023). Some recruitment strategies had costs, and these are also outlined.

**Results:**

A total of 780 potential participants were initially prescreened, 258 completed full REDCap^®^ screening, and 128 people were recruited. Memory clinics/hospitals were the most successful recruitment source, followed by Facebook advertisements and radio.

**Discussion:**

Recruiting people with MCI can be difficult, exacerbated in this study by the concurrent COVID-19 pandemic. Less restrictive inclusion criteria and multisite recruitment may provide greater success.

**Trials registration:**

Australian New Zealand Clinical Trials Registry (ANZCTRN) ACTRN12620001037998. Registered on 12 October 2020.

**Supplementary Information:**

The online version contains supplementary material available at 10.1186/s13063-026-09533-6.

## Background

Mild cognitive impairment (MCI) is a stage between normal cognition and dementia [1]. Prevalence worldwide is suggested to be around 15.6% (95% CI: 13.24%–18.03%) and increases with age [1]. Although research exploring MCI has increased in recent years, much is still unknown about its effect on everyday activities, such as physical function and falls and which treatments are most effective [2].

One in three older people without cognitive impairment fall each year, while for people living with dementia this ranges between 50 and 80% [3]. A recent study by Hopkins and colleagues found that 43% of people living with MCI fall each year, and fall risk factors include slow gait, impaired dual tasking, and postural control [4], suggesting physical function may be starting to decline similar to levels of those with early dementia. Evidence shows that interventions involving functional balance, or a challenge to strength and balance, can significantly reduce falls for people living in the community with no cognitive impairment. There is growing evidence these interventions also assist people living with dementia [5, 6]. However, studies that only include people living with MCI with a focus on falls as the primary outcome are limited [4]. More studies are needed to better understand which interventions are effective for this under-researched population.

Recruitment is another issue for researchers working with people living with MCI. All fall prevention projects involving people living with MCI only (i.e. do not include people with early dementia or subjective cognitive decline) that have been undertaken to date have utilised small sample sizes, and the inclusion criteria for MCI often differs between studies [4]. Also, most trials that exclusively include people living with MCI do not report on their recruitment strategies outside of the main manuscript. This has meant details and issues in recruiting participants and the strategies taken and sources used to recruit are rarely detailed. It is expected that having a better understanding of the successes or failures in different contexts will greatly assist researchers recruiting people exclusively with MCI in future studies; currently, we found two such papers [7, 8]. Sanders and colleagues [7] had greater recruitment success in a nonmedical sport university site than at a memory clinic linked to a hospital, and they recruited across three European countries, whereas Shadyab et al. [8] found mass mailouts were the most successful recruitment source in the USA for the EXERT trial. Both studies completed recruitment prior to COVID-19 and were exploring the effect of exercise on cognition, which is likely more enticing than reducing falls, after a diagnosis of MCI. It must be noted there are many more papers published explaining recruitment strategies and successes for people living with dementia (e.g. [9–13]) or no cognitive impairment (e.g. [14–16]). To assist future intervention studies and for researchers to better understand how previous research has succeeded in recruitment of people with MCI, it is important to publish the recruitment results, both successes and failures. The aim of this study was to determine the effectiveness and cost of different recruitment strategies to recruit people living with MCI into a 12-month non-pharmacological randomised controlled trial aimed at improving balance and reducing falls, known as Balance on the Brain [17].

## Methods

### Overview of Balance on the Brain

Balance on the Brain is a multimodal exercise programme (encompassing balance and walking interventions) aimed at improving balance, physical performance, quality of life, and falls efficacy and reducing falls for people living with MCI [17]. The study protocol has been published [17], and the trial has been registered with the Australian New Zealand Clinical Trials Registry (ACTRN12620001037998). The intervention was a 6-month multimodal exercise programme, which included two balance programmes (i.e. Balance Yourself (a book) and Clock Yourself (an app)). Each intervention participant received six face-to-face home visits and four motivational phone calls across the 6 months. All participants received an educational flyer, and controls were asked to continue with their daily life and usual care. All participants received monthly calls from a blinded research assistant asking about their physical activity levels, health, and whether they had any falls in the previous month. The study was approved by the South Metropolitan Health Service, Western Australia Department of Health, and Curtin University Human Research Ethics Committee prior to any participants consenting to participate. The study was also performed in accordance with the ethical standards as laid down in the 1964 Declaration of Helsinki and its later amendments or comparable ethical standards.

### Sample

The inclusion criteria for participating in the study were as follows: living across metropolitan Perth and Rockingham areas of Western Australia (WA), aged over 50 years, community dwelling (i.e. not in residential aged care), diagnosis of MCI or consistent with the Petersen criteria for MCI, not meeting the Australian physical activity guidelines (i.e. < 150 min of moderate-intensity physical activity per week self-reported), and not participating in at least two balance training sessions per week.

Exclusion criteria included the following: unstable medical condition, terminal illness, diagnosis of significant cognitive impairment or chronic mental illness (e.g. schizophrenia), severe sensory impairment affecting mobility, living in residential aged care, drinking more than 28 standard alcoholic drinks per week; a score of > 6 on Geriatric Depression Scale (GDS) if they have not been diagnosed with a mental health condition, and inability to speak or understand English.

### Recruitment methods

There were 11 different recruitment methods used to recruit participants into the Balance on the Brain study, and each is outlined below. Any written material, such as flyers or newspaper advertisements, were checked by our two consumers (both who were living with memory issues) for language and acceptability prior to use.

1. Memory clinics and hospital physiotherapy and occupational therapy
  In Western Australia, a number of hospitals have memory or cognitive clinics attached to them. Staff (i.e. geriatricians, neurologists, nurses, allied health practitioners) at the following memory clinics assisted with recruitment: Sir Charles Gairdner-Osborne Park Hospital Group, Armadale Hospital, Neurosciences Unit (Graylands Hospital), Rockingham Hospital (physiotherapists working on the wards and outpatient clinics), and Royal Perth Hospital. An occupational therapist at Fremantle Hospital working on the wards also assisted with recruitment. Presentations were given to staff at the memory clinics and to physiotherapists at Rockingham Hospital which provided staff with an opportunity to ask questions about the study and recruitment. Recruitment flyers were also given out at these presentations, which could be used to promote the study. When a potential participant was diagnosed with MCI at one of these hospitals/memory clinics, they were asked if they would like to be contacted about Balance on the Brain, and if they did, their contact details were provided to the research staff for follow-up. Staff were emailed a fortnightly update on recruitment numbers at each site for the whole recruitment time period.
2. GP clinics
  Research staff attended 20 general practice clinics throughout the northern suburbs of Perth and handed promotional flyers about the study to the practice administrators and asked if they could be pinned to the community notice boards. GP clinics were included in late 2022 and 2023 in the recruitment strategy due to slow early recruitment and post-COVID lockdowns.
3. Assessment services
  After gaining clearance from the Federal Government leading aged care across Australia in May 2022, we presented to the Western Australian (WA) Aged Care Assessment Team (ACATs) staff and answered questions about the study and recruitment at one of their monthly meetings. Like the memory clinic staff, if they had patients with cognitive issues but no diagnosis of dementia, they were asked to contact the research staff if a patient was interested in hearing more about the research project. The ACAT team assess older adults for eligibility for Australian aged care services; at the time of recruitment, these were known as the Commonwealth Home Support Program (low-level services such as domestic assistance, gardening, transport), home care packages (higher-level services in the home), or admission to residential aged care.
4. Social media
  Facebook advertisements were published weekly throughout recruitment at a cost of $50 per advertisement. These were targeted at people aged 50 and over and living in Perth and Mandurah-Rockingham areas.
5. Newspapers
  Newspaper articles were included in free community newspapers, and a paid advertisement was included in the leading hard copy newspaper in WA, called The West Australian.
6. Radio
  Curtin FM Radio is the radio station for Curtin University and has a listenership of approximately 55,000 people. Fifteen second advertisements on Curtin FM Radio promoting the research project were undertaken for 3-month periods on multiple occasions across the recruitment period. A Curtin FM radio host also interviewed the lead researcher about the project in a 10-min segment on 6 May 2022. Capital Radio was also contacted about promoting the research programme and provided with a script for the advertisement. It is unclear how many times the advertisement was played by Capital Radio.
7. Website
  A website called www.balanceonthebrain.com was developed and allowed people to read about the project and access the participant information and consent form prior to contacting the research staff if they wished. The website was included on all of the promotional flyers.
8. Dementia registry
  StepUp for Dementia Research (https://www.stepupfordementiaresearch.org.au/) is led by researchers at the University of Sydney and connects people living with a cognitive impairment to researchers conducting research projects throughout Australia [18]. Researchers complete a training session and then are provided with access to the registry and people who meet their study inclusion criteria. Potential participants are then phoned about the study, and it is noted within the registry database as to whether the person was recruited into the study or not.
9. Newsletters
  The Council on the Ageing WA (COTAWA) is the peak body for older people in WA, Injury Matters is a not-for-profit organisation that works to prevent injuries (including falls) and support recovery across WA, the City of Rockingham is a local government, and the Curtin enAble Institute is also involved. Each of these organisations have monthly newsletters which are sent electronically to thousands of members or subscribers. Promotional advertisements were included in a number of these newsletters across the recruitment period.
10. Retirement villages
  Promotional flyers were posted on residents’ noticeboards at Swan Care, Bethanie, and Bullcreek Royal Australian Air Force (RAAF) retirement villages.
11. Presentations
  Promotional presentations were made to the Australian Physiotherapy Association–WA Division (Gerontology) and peer volunteers working with Injury Matters in falls prevention education.

### Data collection

When a person was contacted by the research team as a potential participant, they had the research project explained, before being asked some initial questions to see whether they would be eligible to go through the full pre-screening. The initial pre-screening questions included age, where the person was living, how physically active they were, if they had any issues with their memory, and if they had any medical conditions precluding them from being physically active. If they met these initial pre-screening questions, they then proceeded to the full screening to determine eligibility. The first section of the full screening was undertaken over the phone by a research assistant using REDCap^®^ and took approximately 30–45 min to complete. The final stage of full screening occurred within the home of the potential participant so that the Cognitive Dementia Rating (CDR) and the Standardised Mini-Mental State Examination (SMMSE) could be undertaken face to face. Written consent was provided in person by the person with mild cognitive impairment during this visit. No family members or carers provided proxy consent. The exclusion criteria regarding inability to speak English were based on some aspects of the assessments and intervention needing to be undertaken in English, due to budget constraints impacting use of interpreters and translated resource materials. If a family member was willing to be present during these times and translate to their family member, they were able to participate if they met all other inclusion criteria.

### Data analysis

All potential recruits were initially recorded in an Excel spreadsheet with the date they contacted research staff, their name, contact information, and where they heard about the study (i.e. recruitment source). If they did not proceed to full screening in REDCap^®^, a reason was logged in the Excel spreadsheet next to their name. Those who went through to full REDCap^®^ screening had the recruitment source included in the questionnaire, and if they did not meet the eligibility criteria, the reason why was reported also. Numbers were calculated for each of the 11 recruitment sources at the pre-screening, full REDCap^®^ screening, and those consenting to participate in the intervention. Where recruitment strategies had a cost to the research team/project, they were either reported (i.e. one off newspaper advertisement) or costs combined over the recruitment period (i.e. weekly Facebook advertisements).

Once pre-screening, full REDCap^®^ screening, and consent to participate numbers were confirmed, we calculated the percentage of those who were recruited (S3) by initial pre-screening (S1) and those that were recruited (S3) by full REDCap^®^ screening (S2) by each separate recruitment source (e.g. memory clinics, radio, Facebook). We then used a one-sample *t*-test to compare the mean rates of recruitment across recruitment sources compared to memory clinics (*excluding GP clinics, assessment services, newsletters, and presentations due to zero participants in S3). IBM SPSS version 28 was used for data analysis, and *p*-values < 0.05 were considered statistically significant.

## Results

### Overview of recruitment

**Fig. 1:**
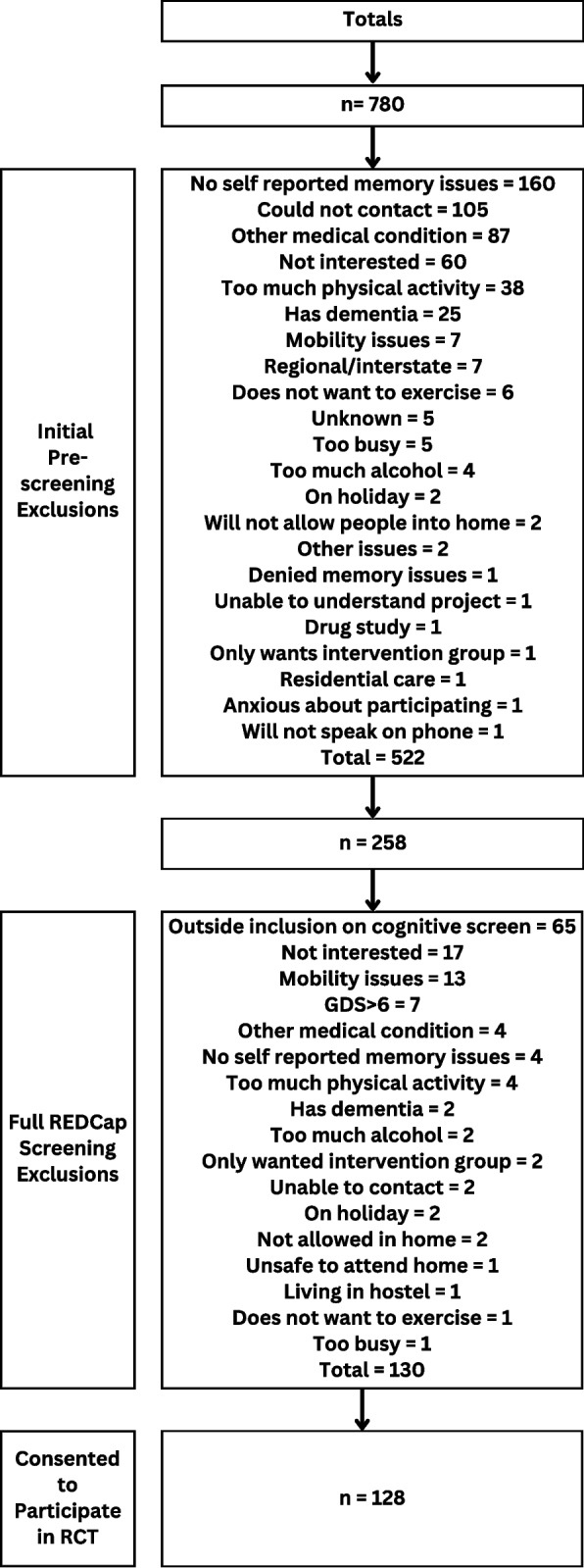
Recruitment flow chart

Recruitment was undertaken from 12 January 2021 to 31 December 2023. Seven hundred and eighty people were asked the initial pre-screening questions over the phone. Five hundred and twenty-two (66.9%) were excluded at that point, and 258 completed the full screening using REDCap^®^ which, for many, took place during their first home visit. After excluding 130 potential participants during the REDCap^®^ full pre-screen, 128 participants (49.6%) were recruited and consented to participate in the study. This was an average of 3.65 participants being recruited per month. Figure 1 provides total numbers for the inclusion and exclusions as well as reasons for exclusion at both initial pre-screening and the REDCap^®^ full pre-screen. Supplementary document 1 provides reasons for exclusion at every recruitment source.

1. Memory clinics
  One hundred and twenty-one people were referred from the memory clinics or by hospital staff who had heard about the study. Thirty people did not meet inclusion criteria after the initial pre-screening, and 91 (75.8%) went through to REDCap^®^ full screen. Fifty-four people (42.2% of the total recruited) were recruited into the study from memory clinics, with an average of 1.5 participants per month.
2. GP clinics
  One person enquired by email about the programme because they had seen a flyer at their local GP clinic. They were unable to be contacted and were not recruited into the study. Zero people (0.0%) were recruited into the study from GP clinics.
3. Assessment services
  Two people were referred from an ACAT assessor in the southern suburbs — Rockingham region. Both went through to REDCap^®^ full pre-screen, but neither was recruited because one was living in a hostel and waiting to move into residential aged care and the other had a CDR > 0.5. Zero people (0.0%) were recruited into the study from ACAT assessors.
4. Social media
  Eighty-four Facebook advertisements were posted between 25 February 2021 and 11 December 2023. The advertisement parameters used to tailor the social media advertising included living within 50+ kilometres of Perth and Rockingham regions and aged 50 years and over. The total cost of all advertisements was AUD$4180. Each advertisement ran for an average of 3–4 days, and the average reach per advertisement was 3640 people. There were 109 clicks (i.e. results) per advertisement on average, at a cost of AUD 0.46 cents per click (on average).
  Four hundred and nine people who contacted the research team via Facebook went through the initial pre-screening: 330 were deemed not to meet the inclusion criteria, and 79 (19.3%) went through to the REDCap® full screen. Thirty-four people (26.6% of the total recruited) were recruited into the study from Facebook advertisements at a cost of AUD$122.94 per participant and an average of almost one participant per month.
5. Newspapers
  Articles in community newspapers were free of charge (six community newspapers, two articles across recruitment), while the one advertisement placed in the West Australian on 1st May 2021 cost AUD$1588.83. A total of 53 people saw an article or advertisement about the study in a newspaper and responded. Nineteen people contacted the research team when they saw an article in a community newspaper describing the study. Of these, 6 did not progress past the initial pre-screening, and 13 (68.4%) progressed to the REDCap^®^ full pre-screen. Thirty-four people contacted the research team after seeing the advertisement in the West Australian (the major newspaper for Western Australians). Twenty-one people did not meet the inclusion criteria after pre-screening, and 13 (61.9%) progressed to the REDCap^®^ full screen. Five people (3.9% of the total recruited) were recruited into the study from community newspapers and six (4.7% of the total recruited) from the advertisement in the West Australian newspaper at a cost of AUD$264.81 per participant. A total of 11 (8.6% of the total recruited) participants were recruited via some kind of newspaper article or advertisement into the full study, fewer than 1 per month.
6. Radio
  The radio advertisements and interviews were provided free of charge and ran for 3 months twice a year. One hundred and nineteen people phoned the lead researcher after they heard the advertisement or interview on Curtin FM Radio. Seventy-three people did not progress past the initial pre-screening, and 46 (38.6%) progressed to the REDCap^®^ full screen. Twenty-one people (16.4% of the total recruited) were recruited into the study from Curtin FM Radio advertisements, fewer than 1 per month. There were no participants from Capital Radio.
7. Website
  The website and domain name cost AUD$855.32 across the 5-year project. The website was created by the lead researcher. Forty-two people who enquired via the Balance on the Brain website went through the initial pre-screening. Only 3 (7.1%) people went through to the REDCap^®^ full screen, with 39 being excluded at the initial pre-screening. One person (0.8% of the total recruited) was recruited into the study from the Balance on the Brain website at a cost of AUD$855.32 for the one participant.
8. Dementia registry
  The StepUp for Dementia Research registry is free, but training must be completed prior to accessing any data. Four people were contacted through the StepUp for Dementia Research registry. Two were excluded at the initial pre-screening due to no self-reported memory issues, and the other person was participating in too much physical activity per week. Two (50%) people went through to the REDCap^®^ full screen: one scored very highly on the TICS-M (score of 39) and was excluded, while the other person was recruited into the study. One person (0.8% of the total recruited) was recruited into the study from the StepUp for Dementia Research registry.
9. Newsletters
  All organisational newsletters were asked to disseminate information about the research project provided to them, and there was no cost for the newsletters. These were placed in multiple newsletters (i.e. four per year). Three people contacted the research team after seeing the promotional flyer in the Injury Matters newsletter, and one (33.3%) of those people progressed through to the REDCap^®^ full screen. No one contacted the research team from the other newsletters used to promote the study. Zero people (0.0%) were recruited into the study from newsletters.
10. Retirement villages
  All flyers were free to disseminate across the retirement villages and were hung on the notice boards for the duration of recruitment (i.e. 36 months). Six people phoned the lead researcher after seeing the study flyers up on the notice board of their retirement village. Four people did not progress past the initial pre-screening, and two (33.3%) progressed through to the REDCap^®^ full screen. Two people (1.6% of the total recruited) were recruited into the study from advertisements placed within retirement villages.
11. Presentations
  Despite delivering two presentations to physiotherapist groups with an interest in cognition, no one contacted the researcher after these had occurred. Zero people (0.0%) were recruited into the study from presentations.
12. Unknown
  Twenty people from unknown recruitment sources were taken through the initial pre-screening, and 5 (25.0%) went on to undertake the REDCap^®^ full screen. Four people (3.1% of the total recruited) were recruited into the study from unknown sources. The reason the sources were unknown was due to these people contacting the research assistant by phone after identifying the study from one of our recruitment sources (e.g. Facebook, flyer), and the research assistant did not complete the data collection section for recruitment source (e.g. for some they could not remember how they found out about the study), meaning we had missing data for these 20 people (i.e. unknown recruitment source).

### Comparison between recruitment sources

Table 1 shows the recruitment sources, the number of people who completed the initial pre-screening (S1), the number of people who completed the REDCap^®^ full screening (S2), and the number that went on to give consent to participate in the study (S3). Compared to those who were excluded at the initial pre-screening (*n* = 121), 45% of people who contacted the researchers from a memory clinic/hospital (*n* = 54) were consented into the project (A: S3/S1). In contrast, 59% of memory clinic/hospital recruits went through to participate (*n* = 54) compared to those that advanced to REDCap^®^ full screening but were excluded at this point (*n* = 91) (B: S3/S2). The percentages for the other recruitment sources are also found in Table 1. The mean percentage of all other recruitment sources other than memory clinic/hospital was 18% at initial pre-screening and 55% at REDCap^®^ full screening. There was a significantly higher proportion of subjects recruited from the memory clinic/hospital than all other sources of recruitment at the initial pre-screening stage (A: *p* < 0.001 (45% vs 18%)).

**Table 1:** Comparison between memory clinic/hospital and other recruitment sources

**Recruitment source**	**Initial pre-screening (S1)**	**Full REDCap**^®^** screening (S2)**	**Consented to participate (S3)**	**A** **%1 (S3/S1)**	**B** **%2 (S3/S2)**
Memory clinic/hospital	121	91	54	**45**	**59**
GP clinics	1	0	0		
Assessment services	2	2	0		
Social media	409	79	34	8	43
Newspapers	53	26	11	21	42
Radio	119	46	21	18	46
Website	42	4	1	2	25
Dementia registry	4	2	1	25	50
Newsletters	3	1	0		
Retirement villages	6	2	2	33	100
Presentations	0	0	0		
Unknown	20	5	4	20	80
Mean* of 2–12				**18**	**55**

A: %1, *p* < 0.001 (45% vs 18%); B: %2, *p* = 0.337 (59% vs 55%)

However, there was no significant difference between the memory clinics compared to all other recruitment sources at the REDCap^®^ full screening for those who went through to be consented to participate (B: *p* = 0.337 (59% vs 55%)).

## Discussion

In the Balance on the Brain trial, in which we aimed to test a multi-modal exercise intervention to improve balance and reduce falls for people living with MCI, recruitment of participants was challenging. Building relationships with geriatricians and allied health staff working in memory clinics and hospitals was valuable, with memory clinics being the most effective source of recruitment for this study. This differed from Sanders and colleagues [7] who had more success recruiting people living with MCI into their multi-site 12-month study called NeuroExercise from the community (*n* = 110; recruitment sources: Nijmegen: active ageing interventions already in progress; Dublin: GP practices and radio interviews; Cologne: social media) compared to memory clinics (*n* = 67). They had a total of 177 participants recruited into their study over 18 months, an average of almost 10 participants per month [7].

Shadyab et al. [8] only included adults with amnestic MCI into their multi-site randomised control trial, called EXERT. Recruitment lasted 42 months and included mass mailouts to 490,323 potential participants at a success rate of 52% compared to memory clinics and hospital registers (25% success) [8]. Community sources of recruitment included word of mouth (6.1%), news media (1.7%), community presentations (5.4%), paid advertising (5.4%), and other (< 2%) [8]. In total, they screened 992 people with 296 randomised into the trial across 14 sites [8], an average of 7 participants per month. Both of these studies reported higher recruitment rates compared to the Balance on the Brain trial, with an overall recruitment rate of almost 4 participants per month, compared to 10 with NeuroExercise and 7 with EXERT. It is recommended that studies recruiting people living with MCI only and no other cognitive impairment (e.g. dementia) or people with no cognitive impairment are conducted across multiple sites or multi-countries to maximise exposure to this population, which can be challenging to engage. Also, the Balance on the Brain trial did not attempt any mass mailouts, and this potentially may have been a successful strategy as was the case for the EXERT trial [8].

Limitations of recruitment for this current study included the fact that the majority of the recruitment period occurred during the COVID-19 pandemic. Although Western Australia did not experience the long lockdowns of states such as Victoria in Australia, at least 8 weeks were lost to recruitment during this time period (between March and July 2021), and daily public health messaging by government (Federal and State) encouraging older people to limit human contact meant many were concerned about people coming into their homes due to the risk of infection throughout much of the recruitment period. The Sanders et al. [7] and Shadyab et al. [8] studies both completed recruitment prior to COVID-19.

Although difficult to trace, the recruitment team did notice it became more difficult to contact potential participants over the phone or via email in 2022 and for the remainder of the recruitment period. A possible contributory factor for this may have been because they were afraid of being scammed. We hypothesise this to be an issue because our monthly phone calls across the 12 months to all participants became more difficult across the study duration; we asked participants why they were not answering their phones, and they commonly stated they were worried about being scammed. Scamming and warnings about scammers on news networks in Western Australia increased markedly during COVID-19 and during 2021. For example, there was a 43% increase in the number of people contacting IDCARE’s national scam and cyber-crime victim support service in Australia in 2021 compared to the previous year [19]. News programmes in Western Australia also reported Australians being “easy targets” for scammers, and that during the height of COVID-19, Australia was placed in the top 4 countries in the world being targeted by international scammers [19]. This likely reduced our recruitment success, particularly in the latter stages of recruitment. As more time passes since the height of the COVID-19 pandemic, we have noticed in other studies that participants are now answering the phone more often again, and segments on scamming on the news have also reduced. The restrictions on face-to-face recruitment strategies during COVID-19 would also likely be more successful for future researchers as they were for those prior to the pandemic. Therefore, researchers should be encouraged to use these strategies more also.

It must also be noted that staff time to develop the resources (e.g. website, flyers) and recruit participants was not collected separately from the screening or recruitment processes. Therefore, the costs provided are all non-staff resources, and researchers looking to recruit this population need to factor staffing costs into their recruitment strategies also. The analysis across the different types of recruitment also has limitations because it was not possible to factor in the different types of recruitment, such as passive (flyers) versus active (recommended by a geriatrician); therefore, these results should be interpreted with caution.

Our recruitment criteria outside of the MCI diagnosis were quite restrictive, and in future studies, it may be beneficial to have fewer restrictions on inclusion criteria, for example not meeting the physical activity guidelines and balance training weekly could be changed to not participating in any or limited balance training. As mentioned earlier, including multiple sites would likely have assisted with boosting recruitment also. Also, not having a budget for translators meant people who spoke a language other than English were not included unless a family member was willing to participate in monthly phone calls and home visits for intervention participants.

GPs were also a factor in Balance on the Brain where recruitment was not successful, especially compared to the memory clinics and hospital recruitment. This may be due to the length of appointments and administrative staff support. A routine memory clinic model of care includes longer appointments (45 min new patient, 30 min routine reviews) compared to GPs (15 min standard Medicare rebate). In a 15-min GP appointment, doctors are often not resourced to give additional time and other adjustments for people with cognitive impairment. Therefore, it may be the accessibility of information and lack of time to explain the project that was a major limitation for the GPs involved. Flyers on the notice boards at GP clinics also were not an effective recruitment strategy. Additionally, Balance on the Brain had an intervention duration with people asked to participate for 12 months, and those receiving the intervention had physiotherapist visits for 6 months. This long period of commitment to the study may have also deterred a number of people from contacting the research team. However, given the current dearth of fall prevention intervention studies exclusively for people living with MCI (*n* = 2) and that they have had small sample sizes (e.g. *n* = 23 per group) or only included falls as adverse events and not a primary outcome, Balance on the Brain will improve our understanding on falls and balance for people living with MCI over a 12-month period and assist future research for this important cohort. Main study results are in preparation and expected to be available in the near future and will include participant characteristics.

Recruitment into Balance on the Brain was challenging, and a number of recruitment sources were used to bolster recruitment, with the most successful being memory clinics, Facebook advertisements, and radio. Our findings suggest it is necessary to utilise many different options to recruit people living with MCI into long-term studies, and using multiple sites is likely to be more successful. Mass mailouts should also be considered for this population when conducting nonpharmacological trials.

## Supplementary Information

Supplementary Material 1. Figure 1. Recruitment flow chart by recruitment strategy

## Data Availability

Please contact the lead author should you wish to request data from this study.

## Abbreviations

ACAT: Aged Care Assessment Team
CDR: Cognitive dementia rating
COTAWA: Council on the Ageing Western Australia
MCI: Mild cognitive impairment
SMMSE: Standardised Mini-Mental State Examination
WA: Western Australia
