# Supplementary material for: Recruiting people living with mild cognitive impairment into a fall prevention trial
Source: Trials. 2026 Feb 10;27:154. doi: 10.1186/s13063-026-09533-6 (PMC12918016; doi:10.1186/s13063-026-09533-6)
Supplement: Supplementary file 1 — Supplementary Material 1. Figure 1. Recruitment flow chart by recruitment strategy [file 13063_2026_9533_MOESM1_ESM.pdf]

Supplementary Figure 1. Recruitment flow chart by recruitment strategy (Page 1 of 2)

	**Memory Clinic**	**GP Clinic**	**Assessment Clinic**	**Social Media**	**Newspapers**	**Radio**
	n=121	n=1	n=2	n=409	n=53	n=119
**Initial Pre-screening Exclusions**	Not interested = 12<br>Could not contact = 7<br>Other medical condition = 3<br>On holiday = 2<br>Denied memory issues = 1<br>Unable to understand project = 1<br>Too much alcohol = 1<br>Drug study = 1<br>Unknown = 1<br>Only wants intervention group = 1<br>Total = 30	Could not contact after sending return email = 1<br>Total = 1		No self reported memory issues = 88<br>Could not contact = 83<br>Other medical condition = 60<br>Not interested = 42<br>Too much physical activity = 23<br>Mobility issues = 6<br>Does not want to exercise = 6<br>Has dementia = 6<br>Regional/interstate = 5<br>Too busy = 4<br>Will not allow anyone into their home = 2<br>Moving into residential aged care = 1<br>Other issues = 1<br>Anxious about participating = 1<br>Too much alcohol = 1<br>Will not speak on phone = 1<br>Total = 330	Community News<br>No self reported memory issues = 3<br>Not interested = 2<br>Unknown = 1<br>Total = 6<br><br>The West Advert<br>No self reported memory issues = 11<br>Other medical condition = 4<br>Has dementia = 4<br>Unknown = 1<br>Too much physical activity = 1<br>Total = 21<br><br>Combined total = 27	No self reported memory issues = 35<br>Other medical condition = 13<br>Too much physical activity = 9<br>Has dementia = 8<br>Could not contact = 4<br>Unknown = 2<br>Too much alcohol = 1<br>Mobility issues = 1<br>Total = 73
	n=91	n=0	n=2	n=79	Community news = 13<br>The West advert = 13<br>Total = 26	n=46
**Full REDCAP Screening Exclusions**	Not interested = 10<br>Mobility issues = 9<br>Outside inclusion on a cognitive screen = 8<br>GDS>6 = 2<br>Has dementia = 2<br>Too much alcohol = 1<br>No self reported memory Issues = 1<br>Only wants intervention group = 1<br>Unsafe to attend home = 1<br>Unable to contact = 1<br>Too much physical activity = 1<br>Total = 37	Total = 0	Living in a hostel = 1<br>CDR >0.5 = 1<br>Total = 2	Outside inclusion on cognitive screen = 29<br>Not interested = 5<br>Other medical condition = 3<br>GDS>6 = 2<br>Mobility issues = 2<br>Not available (holidaying) = 2<br>Not allowed in home = 1<br>Too much PA = 1<br>Total = 45	Community News<br>Outside inclusion on cognitive screen = 5<br>GDS>6 = 1<br>Only wants intervention group = 1<br>Does not want to exercise = 1<br>Total = 8<br><br>The West Advert<br>Outside inclusion on cognitive screen = 5<br>Mobility issues = 1<br>Too busy = 1<br>Total = 7<br><br>Combined total = 15	Outside inclusion on cognitive screen = 15<br>GDS>6 = 2<br>Too much PA = 2<br>Other medical condition = 1<br>Not allowed in home = 1<br>Not interested = 1<br>Could not contact = 1<br>Mobility issues = 1<br>Too much alcohol = 1<br>Total = 25
**Consented to Participate in RCT**	n=54	n=0	n=0	n=34	Community news = 5<br>The West advert = 6<br>Total = 11	n=21

Supplementary Figure 1. Recruitment flow chart by recruitment strategy (Page 2 of 2)

	**Website**	**Dementia Registry**	**Newsletters**	**Retirement Villages**	**Presentations**	**Unknown**
	n=42	n=4	n=3	n=6	n=0	n=20
**Initial Pre-screening Exclusions**	No self reported memory issues = 14<br>Could not contact = 7<br>Other medical condition = 5<br>Has dementia = 4<br>Not interested = 3<br>Too much physical activity = 2<br>Regional/interstate = 2<br>Spouse died = 1<br>Total = 38	No self reported memory issues = 1<br>Too much physical activity = 1<br>Total = 2	No self reported memory issues = 1<br>Other medical condition = 1<br>Total = 2	No self reported memory issues = 1<br>Other medical condition = 1<br>Has dementia = 1<br>Too much PA = 1<br>Total = 4	Total = 0	No self reported memory issues = 6<br>Could not contact = 3<br>Has dementia = 2<br>Too busy = 1<br>Not interested = 1<br>Too much alcohol = 1<br>Too much physical activity = 1<br>Total = 15
	n=4	n=2	n=1	n=2	n=0	n=5
**Full REDCAP Screening Exclusions**	Not interested = 1<br>No self reported memory issues = 2<br>Total = 3	TICS-M too high = 1<br>Total = 1	TICS-M too high = 1<br>Total = 1	Total = 0	Total = 0	No self reported memory issues = 1
**Consented to Participate in RCT**	n=1	n=1	n=0	n=2	n=0	n=4
